# An Overview of the Presence of Cephalosporin Antibiotics in Aquatic Environments

**DOI:** 10.3390/ph19040650

**Published:** 2026-04-21

**Authors:** Ramona-Alexandra Ciausu, Mircea Nicusor Nicoara, Ionut-Alexandru Chelaru, Gabriel Andrei Andronic, Alin Stelian Ciobica, Dorel Ureche

**Affiliations:** 1 Doctoral School of Geosciences, Faculty of Geography and Geology, “Alexandru Ioan Cuza” University of Iasi, Bd. Carol I, 700505 Iasi, Romania; ciausuramona05@gmail.com (R.-A.C.); chelaru.alexandru@yahoo.com (I.-A.C.); 2 Department of Biology, Faculty of Biology, “Alexandru Ioan Cuza” University of Iasi, Bd. Carol I, 700505 Iasi, Romania; alin.ciobica@uaic.ro; 3 Department of Biology, Faculty of Sciences, “Vasile Alecsandri” University of Bacau, Marasesti Street, 600115 Bacau, Romania; andreigabrielandronic@gmail.com (G.A.A.); dureche@ub.ro (D.U.); 4 “Ioan Haulica” Institute, Apollonia University, Pacurari Street 11, 700511 Iasi, Romania; 5 “Olga Necrasov” Center, Department of Biomedical Research, Romanian Academy, 010071 Iasi, Romania; 6 Multidisciplinary Medical Research and Development Platform in the North-East Region (CENEMED), “Grigore T. Popa” University of Medicine and Pharmacy of Iasi, 700115 Iasi, Romania

**Keywords:** cephalosporin antibiotics, aquatic environments, ecotoxicological effects, antibiotic resistance

## Abstract

**Background:** Cephalosporins, widely used β-lactam antibiotics, are becoming significant environmental pollutants, primarily due to their high use and persistence. They are released into the environment mainly through wastewater treatment plants, agricultural runoff, and hospital discharge, with particularly high concentrations recorded in effluents. Conventional wastewater treatment methods have inadequate removal efficiency, while advanced treatments, such as ozonation, activated carbon adsorption, and advanced oxidation processes, although more efficient, may produce toxic by-products. Recent studies emphasize the importance of improved detection and monitoring techniques and advocate for stricter effluent regulations. Despite growing research attention, important knowledge gaps remain, including limited long-term field monitoring, insufficient data on environmentally realistic exposure scenarios, and incomplete assessment of transformation-product toxicity. **Methods:** The search strategy used the *SCOPUS* and *PUBMED* databases with the keywords “cephalosporin” AND “aquatic environment”, resulting in 341 records. After applying predefined inclusion and exclusion criteria, 110 peer-reviewed English-language studies meeting predefined thematic inclusion criteria and relevant to the occurrence, environmental fate, ecotoxicological effects, antimicrobial resistance, and removal of cephalosporins in aquatic environments were included in the narrative synthesis. **Results:** The literature on cephalosporins in aquatic environments has expanded significantly from 1978 to 2025, prompted by concerns about pharmaceutical contamination and antibiotic resistance. Studies from 2016 to 2025 used advanced and multidisciplinary monitoring techniques, revealed key pollution sources such as wastewater treatment plants and hospitals, and correlated antibiotic residues with resistance genes, highlighting the need for continued monitoring and mitigation efforts. Ecotoxicological and fate studies further indicate that transformation processes may generate products with altered or increased toxicity, complicating environmental risk assessment. **Conclusions:** The literature shows increasing attention to cephalosporins in aquatic environments, reporting associations with antimicrobial resistance and adverse effects on aquatic organisms, including potential toxicity from transformation products. This review highlights the need for integrated monitoring, standardized toxicity assessment, and improved treatment strategies within a One Health framework.

## 1. Introduction

The extensive application of antibiotics in human and veterinary medicine has led to their persistent release into natural ecosystems, raising concerns about ecological and public health impacts [1,2,3]. Despite treatment procedures, antibiotics frequently persist in aquatic environments, facilitating the spread of antibiotic resistance genes and altering microbial populations [4,5]. Various pathways lead to environmental pollution, such as effluents from wastewater treatment plants, agricultural runoff, hospital discharges and landfill leachate, underscoring the complex interactions of antibiotics in aquatic environments [6,7,8]. Antibiotics (ABs) primarily enter aquatic habitats via wastewater treatment plants (WWTPs), where traditional treatment techniques are frequently insufficient to completely remove these compounds. As a result, large amounts of unmetabolized ABs and their transformation products are discharged into receiving surface waters via treated effluents, adding to their ongoing presence and potential ecological impact [9].

Other sources include agricultural runoff, hospital effluents, and landfill leachate [10,11,12]. Studies have shown that tertiary processes have varying degrees of success in removing ABs, with manufacturing facilities being a major point source [9]. In some Asian countries, AB concentrations in effluents are several times higher than in surface waters, indicating that manufacturing facilities may be a major point source of ABs [7,9]. These substances can be measured in aquatic habitats, including surface water, wastewater, sediments and aquatic organisms [6,13]. Laboratory studies have been performed to model key processes such as degradation, adsorption, oxidation and sophisticated treatment approaches, with the aim of improving the understanding of the environmental behavior, transformation pathways and final fate of these compounds. These controlled tests provide significant information on the pharmacokinetics, persistence and elimination efficiency of cephalosporins under various environmental and artificial circumstances [14,15].

Cephalosporins, broad-spectrum β-lactam ABs [15,16], are emerging pollutants due to their widespread use and persistence in environmental matrices [14]. Beta-lactam ABs are active against various Gram-positive, Gram-negative and anaerobic organisms, interfering with cell wall synthesis [17]. They represent the second most prescribed class of ABs in Europe [18]. Although broader classes of ABs are receiving more attention [19], β-lactam ABs, including cephalosporins, are often inadequately documented in environmental research due to their intrinsic chemical instability. The β-lactam ring is highly vulnerable to hydrolysis, especially under conditions of neutral-to=alkaline pH, high temperatures and light. This structural weakness can cause rapid degradation in aquatic environments, as well as during sample collection or storage, resulting in an underestimation of environmental concentrations [6,20]. Cephalosporins were isolated from the fermentation products of *Cephalosporium acremonicum* [21,22]. The main component is cephalosporin C, an amide with *α*-aminoadipic acid and 7-aminocephalosporanic acid. Semisynthetic beta-lactam cephalosporin ABs were created via acylation of the amino group with various acid derivatives. There are approximately 25,000 such ABs, of which approximately 100 are used in medicine [16]. Semisynthetic cephalosporins are synthesized by expanding the acid spectrum and internal modifications of the aminocephalosporanic nucleus [22].

To provide a more comprehensive understanding of the classification of cephalosporins in terms of antibacterial activity and range of action, a summary table detailing the main generations of cephalosporins and their main pharmacological characteristics has been created. This classification, based on Vardanyan and Hruby (2006), helps to better understand the structural evolution and therapeutic relevance of cephalosporins over the generations (Table 1) [22].

Thus, due to their broad spectrum of antibiotic activity and their widespread use in human and veterinary medicine, cephalosporins and their metabolites are released into the environment after consumption and are increasingly found in aquatic compartments. Numerous studies have reported their presence in surface waters, wastewater, sediments and, in some cases, drinking water, indicating their significance and persistence in the environment [6]. Concentrations range from ng L^−1^ in rivers and lakes to mg L^−1^ near pharmaceutical production facilities [7]. Hospital effluents and agricultural runoff contribute significantly to this phenomenon. Over 50 investigations have reported the presence of cephalosporins, with detection being improved by advances in liquid chromatography-mass spectrometry (LC-MS/MS) and high-performance liquid chromatography (HPLC) [27,28]. Novel extraction approaches, such as solid-phase microextraction, have further increased the sensitivity of detection [29].

Wastewater contains toxic compounds and active pharmaceutical ingredients, which pose a threat to the environment [30]. With reported values ranging from 0.3 ng L^−1^ to 0.03 mg L^−1^, cephalosporins, including cephalexin, cefotaxime and ceftriaxone, have been found in surface water, groundwater and influents/effluents across multiple regions worldwide, particularly in Europe and Asia, as reviewed by Ribeiro et al. [15]. In hospital wastewater, cefepime reached concentrations of up to 540 µg L^−1^ (fourth generation) [31], compared to ~8.5 µg L^−1^ in hospital wastewater from Romania [32], indicating marked geographical and usage pattern variability. The removal of cephalosporin ABs is necessary due to increased chemical oxygen demand and the production of cephalosporin-resistant bacteria [14].

Manufacturing facilities in Asia, particularly India and China, report extremely high concentrations of cephalosporins in effluents. Levels of up to mg L^−1^ have been observed, creating strong foci of selection for resistance genes [7]. These point sources highlight the urgent need for stricter regulation and monitoring of effluents in pharmaceutical manufacturing regions. Conventional WWTPs only partially remove cephalosporins, with removal efficiencies typically below 50% [8]. On the other hand, advanced treatment technologies, including ozonation [33], UV/chlorine, advanced oxidation processes (AOPs), activated carbon adsorption and membrane filtration, achieve higher removal rates. However, these treatment processes can produce harmful intermediate compounds as unintended by-products [8], and current evidence suggests that integrated treatment strategies–combining physical, chemical and biological approaches–offer the most effective and sustainable solution for reducing pharmaceutical contamination.

In aquatic environments, cephalosporins undergo hydrolysis, photolysis and biodegradation [34]. Furthermore, studies on phototransformation reveal half-lives ranging from hours to days and are influenced by organic matter, light and pH. The rate of hydrolysis is faster in alkaline environments. According to Pruden et al. (2006), sediments frequently serve as sorbents, delaying deterioration [35]. A crucial aspect is that transformation products may still be biologically active or more harmful than the parent chemicals, which requires careful monitoring. Analytical chemistry has advanced rapidly, allowing the detection of traces at ng L^−1^ levels [28]. Modern LC-MS/MS and high-throughput methods allow the simultaneous detection of multiple ABs. Recent innovations also include robust workflows for sample preparation and the application of quality by design principles for method validation [36]. These tools improve the reliability of monitoring programs and regulatory compliance. Policy-oriented studies emphasize risk assessment, mitigation strategies, and “One-Health” frameworks. Recommendations include stricter limits for effluent discharges, systematic monitoring of influent/effluent from WWTPs, and the integration of antimicrobial resistance (AMR) considerations into water quality standards [37]. This study aims to summarize and critically synthesize current findings on the occurrence, environmental fate, ecotoxicological effects, antimicrobial resistance associations, and removal of cephalosporins in aquatic environments.

### Novelty and Scope of the Review

Several reviews have previously addressed the environmental occurrence of antibiotics as emerging contaminants; however, most considered cephalosporins only as part of broader antibiotic classes or within geographically limited case studies. For example, Das et al. (2019) provided an early overview of cephalosporins as environmental pollutants, primarily focusing on occurrence concerns, while lacking systematic synthesis of transformation pathways, ecotoxicological effects, and antimicrobial resistance implications [14]. Similarly, Mirzaei et al. (2017) investigated the occurrence and fate of commonly prescribed antibiotics in specific water environments in Iran, without a cephalosporin-centered analytical or mechanistic perspective [38].

In contrast, the present review offers a comprehensive and cephalosporin-specific synthesis, systematically integrating data from 110 peer-reviewed studies published between 1978 and 2025. This work uniquely combines information on compound-specific occurrence, analytical detection, environmental fate and transformation products, ecotoxicological impacts, antimicrobial resistance associations, and treatment/removal technologies, framed within a One Health perspective [39]. By focusing exclusively on cephalosporins and incorporating recent advances in monitoring, degradation processes, and resistance research, this review addresses critical knowledge gaps and provides a more integrative foundation for environmental risk assessment and regulatory decision-making.

## 2. Materials and Methods

The literature search was conducted using the SCOPUS and PUBMED databases with the keywords “cephalosporin” AND “aquatic environment”. The search was restricted to peer-reviewed articles published in English, with no geographical limitations.

**Figure 1:**
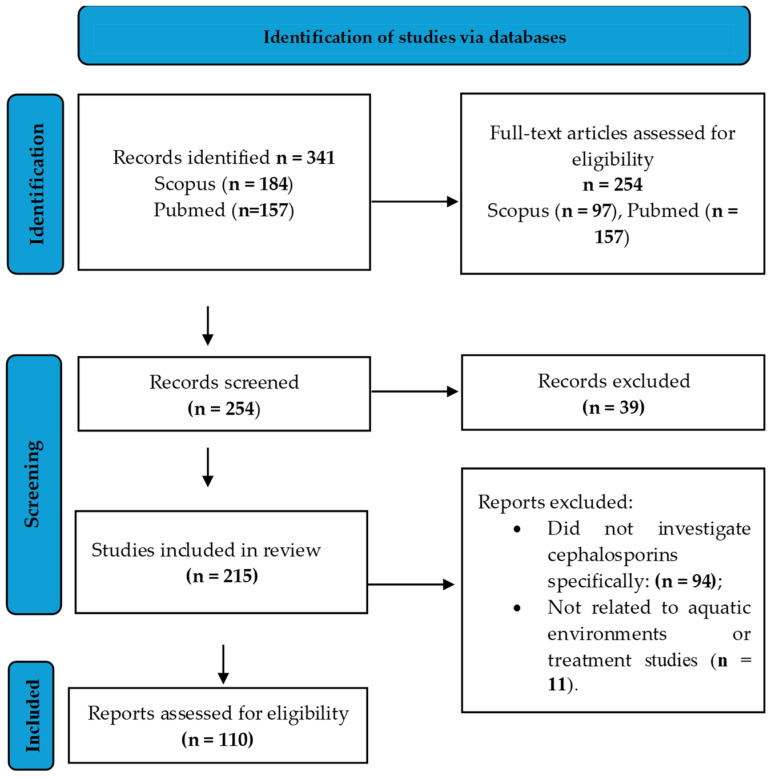
Prisma Flow Diagram [40].

A total of 341 records were identified by the database search (Figure 1). For Scopus, 184 results were found and 97 were selected from the research areas Environmental Science and Pharmacology, Toxicology and Pharmaceuticals. The search was restricted to English. For PubMed, 157 results were found. After removing 39 duplicates between databases, there were 215 unique records for screening titles and abstracts. During this screening stage, studies were assessed based on their relevance to cephalosporins in aquatic environments, including at least one of the following aspects: occurrence and monitoring, analytical determination, environmental fate and degradation, ecotoxicological effects, antimicrobial resistance, or removal/treatment processes. Although several studies investigated multiple antibiotic classes simultaneously, only cephalosporin-specific data were extracted and synthesized in this review, ensuring a compound-focused analysis.

The excluded reports (n = 105) were classified as follows:

- cephalosporins were not specifically investigated (n = 94): research focused on generic ABs, antimicrobial resistance, or other pharmacological substances.
- not related to aquatic environments or treatment studies (n = 11): studies that refer to cephalosporins but do not include environmental, analytical, or aquatic components.

The narrative synthesis presented in this review was based on the final set of 110 papers.

## 3. Results and Discussion

**Figure 2:**
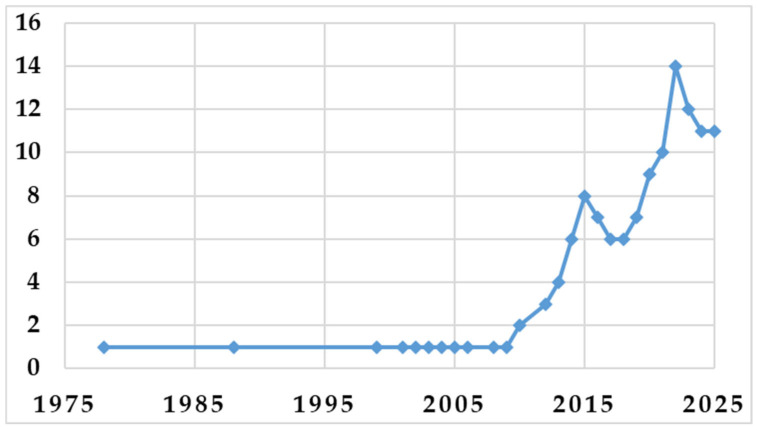
Annual Scientific Production.

The annual scientific output on cephalosporins in aquatic environments has been steadily increasing, indicating a growing global concern about pharmaceutical contamination and antibiotic resistance in water systems. The 110 eligible studies, published between 1978 and 2025, reflect a sustained and evolving research interest in cephalosporins in aquatic and environmental contexts (Figure 2). Early publications from 1978 to 1999 were limited and focused primarily on microbiological and clinical issues with indirect implications for the environment, including patterns of susceptibility and resistance in aquatic isolates. From 2004 to 2009, research increasingly focused on environmental exposure and ecological risks related to antibiotic use, indicating a shift towards greater environmental awareness. Since 2010, there has been a significant increase in the number of publications, in parallel with the growing recognition of pharmaceuticals as emerging environmental contaminants. Between 2010 and 2015, research shifted to antimicrobial resistance in aquatic systems and the environmental consequences of antibiotic use.

Most of the included papers were published between 2016 and 2025, when scientific productivity increased most significantly. This period is distinguished by the widespread monitoring of cephalosporins in surface waters and wastewaters, laboratory investigations of fate and degradation, development of analytical techniques, and evaluation of treatment and removal strategies. The temporal pattern indicates a transition to integrated, multidisciplinary approaches that include environmental chemistry, toxicology, and microbiology.

Overall, the upward trajectory demonstrates a rapidly expanding field of study, with continued scientific interest and increasing identification of cephalosporins as emerging aquatic pollutants requiring ongoing monitoring and mitigation efforts. In parallel, the development and refinement of modern analytical and detection technologies have further accelerated research productivity, allowing the identification of contaminants at ever lower concentrations, and supporting a broader scientific understanding of their behavior in the environment.

**Figure 3:**
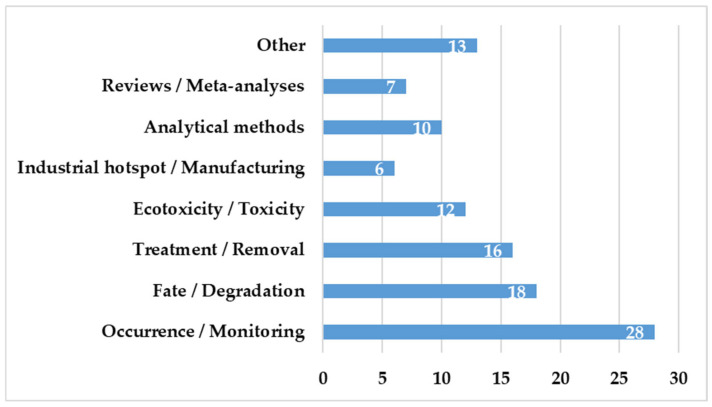
Thematic Classification of Included Studies (N = 110).

The papers included in this review were divided into broad thematic groups, based on the main objective of the study (Figure 3). The largest group consisted of occurrence and monitoring studies, which demonstrated a continuing interest in the detection and quantification of cephalosporins in aquatic environments, such as surface waters, wastewaters, sediments, and biota. These investigations typically used advanced analytical techniques, such as LC-MS/MS, to determine geographic and temporal contamination patterns. Research on the Fate and Degradation of cephalosporins investigated their transformation under environmental and simulated laboratory circumstances, such as photolysis, hydrolysis, and oxidation processes, and frequently identified transformation products and reaction pathways. The Treatment and Removal category included research that evaluated traditional and advanced treatment strategies, such as adsorption, electrochemical degradation, and advanced oxidation processes, to reduce cephalosporin concentrations in water and wastewater matrices.

Ecotoxicity/toxicity studies investigated the biological effects of cephalosporins and their transformation products on aquatic species, reporting consequences at different trophic levels and biological endpoints. A smaller but significant subset of studies has focused on industrial hotspots and pharmaceutical manufacturing, with pharmaceutical effluents identified as key point sources of cephalosporins in aquatic habitats. The Analytical Methods category included studies on the development, optimization, and validation of analytical methodologies for the detection of cephalosporins at trace levels in complex environmental matrices. Finally, Reviews and Meta-Analyses summarized current knowledge on incidence, fate, toxicity, and removal options, while other works focused on developing areas such as antimicrobial resistance genes, interactions with microplastics, and environmental modeling.

### 3.1. Occurrence and Analytical Assessment of Cephalosporins in Aquatic Environments

Analytical advances have enabled the sensitive detection of cephalosporins and other ABs in a variety of aquatic matrices, with LC-MS/MS and high-resolution mass spectrometry becoming the dominant approaches [27,28]. ABs can be found in surface waters, groundwater, sediments and wastewater at trace or low concentrations (µg L^−1^), [6,15]. Due to the intrinsic instability of the β-lactam ring, reported environmental concentrations of cephalosporins may be underestimated when sampling, storage, and stabilization protocols are not consistently applied [15]. Wastewater treatment plants and hospital effluents are major sources, and inadequate disposal leads to continued discharge into receiving waters [8,32]. Unlike other antibiotic classes commonly used in aquaculture, cephalosporins are not widely approved for routine aquaculture applications, and their detection in aquaculture-impacted waters is therefore more plausibly attributed to human, hospital, and veterinary sources rather than direct aquaculture use. Several studies have found strong links between antibiotic residues and antibiotic resistance genes or multidrug-resistant bacteria, highlighting the implications of cephalosporin contamination on the environment and public health [5,35].

Table 2 presents the analytical methodologies and occurrence studies demonstrating the presence of cephalosporins and other ABs in aquatic environments. The studies in this table show the widespread use of advanced chromatographic techniques, in particular solid phase extraction (SPE) coupled with LC-MS/MS and high-resolution mass spectrometry (HRMS), for the detection and quantification of cephalosporins at trace levels [27,28,41]. Environmental matrices investigated include surface waters, groundwater, wastewater influents and effluents, sediments and pond water.

ABs are consistently detected in aquatic systems affected by urban, hospital and industrial activity, with sewage treatment plants being recognized as major sources of environmental pollution. Several studies have also found that ABs are not completely removed during typical wastewater treatment techniques, allowing them to persist in receiving water bodies. In addition, several studies show associations between antibiotic incidence and the availability of antibiotic resistance genes or multidrug-resistant bacteria, highlighting the environmental importance of cephalosporin contamination [7,35]. Overall, the research summarized in Table 2 provides strong evidence of the extensive use of cephalosporins in aquatic ecosystems, emphasizing the importance of rigorous analytical methodologies for environmental monitoring and risk assessment.

It should also be noted that aquatic organisms are typically exposed to mixtures of antibiotics and co-occurring pollutants; however, mixture effects involving cephalosporins remain poorly characterized and represent an important direction for future ecotoxicological research.

### 3.2. Ecotoxicological Effects of Cephalosporins

The ecotoxicological effects of cephalosporins and their transformation products have been evaluated in a variety of aquatic animals such as invertebrates and fish, and also microorganisms and primary producers, taking into account numerous trophic levels and biological endpoints (Table 3). Several studies have examined acute and chronic toxicity to aquatic invertebrates, particularly *Daphnia magna*. The ecotoxicity of the veterinary cephalosporins ceftiofur and cefapirin has been studied before and after phototransformation, revealing that the photoproducts may have different toxicity profiles from the parent compounds, with effects on immobilization and growth inhibition in D. magna and Lemna minor [49]. Similarly, chronic exposure of *D. magna* to cefadroxil and cefradine had detectable impacts on survival, reproduction, and growth, indicating possible long-term ecological concerns at relevant doses [50].

Behavioral endpoints were investigated using rotifers (*Brachionus calyciflorus*), and exposure to ceftazidime and its photoproducts significantly affected feeding rates and post-exposure recovery, demonstrating that sublethal behavioral changes can serve as sensitive indicators of cephalosporin toxicity [51]. Cephalosporin exposure has also been found to affect primary producers. The green microalga *Chlorella vulgaris* showed growth suppression and reduced photosynthetic activity when exposed to cephalexin; however, adaptive responses were observed under certain exposure scenarios, implying species-specific tolerance mechanisms [52]. Another work used *Chlorella* sp. both as a biological therapy agent and as a toxicity receptor, demonstrating that removal of ceftazidime by algae resulted in quantifiable biochemical and physiological stress responses [53].

Fish models have provided insights into higher-level biological impacts. A study by Xue et al. (2023) using zebrafish (*Danio rerio*) demonstrated that environmentally realistic concentrations of cefotaxime increased the mobility of β-lactam resistance genes in the gut microbiota [54]. This highlights indirect ecological risks related to the spread of antimicrobial resistance, rather than classical toxicity endpoints. However, studies directly addressing immune responses or broader gut microbiome alterations in fish exposed to cephalosporins remain scarce, highlighting an important knowledge gap rather than a lack of ecological relevance. Moreover, the attribution of observed resistance gene mobility specifically to cephalosporins remains uncertain, as co-selective agents and complex environmental mixtures may also contribute to these effects [54].

Effects of chronic exposure have also been observed in medaka fish (*Oryzias latipes*), including changes in growth and survival after exposure to cefadroxil and cefradine [50]. Several studies have highlighted the importance of transformation mechanisms in the regulation of toxicity. Photochemical degradation of cephalosporins affected their acute toxicity to *D. magna*, with several photoproducts demonstrating higher or persistent toxicity despite significant elimination of the parent compound [55]. Similarly, ionizing radiation-induced degradation of cephalosporin C produced transformation products with detectable toxicity in immobilization studies with *D. magna* [55]. At the microbiological level, chlorination of cefazolin produced transformation products capable of triggering DNA damage in *Escherichia coli*, as demonstrated by genotoxicity studies, raising concerns about disinfection byproducts in treated waters [56].

Finally, computational approaches have complemented experimental studies. A theoretical study by Masmoudi et al. (2022) on hydroxyl radical-triggered degradation of cephalexin suggested that various transformation products could retain or increase ecotoxicological potential, highlighting the importance of evaluating degradation byproducts alongside parent molecules [57].

Overall, these investigations show that cephalosporins can have a variety of biological effects on aquatic organisms, with toxicity being determined by the structure of the compound, duration of exposure, environmental transformation processes, and biological complexity. It is important to note that numerous studies show that degradation or treatment methods do not always eliminate environmental hazards and may, in some situations, result in compounds with altered or increased toxicity.

**Table 3:** Ecotoxicological Effects of Cephalosporins on Aquatic Organisms.

Cephalosporin	Organisms	Effects	Concentration	Exposure	Country	References
Ceftiofur, Cefapirin	*Daphnia magna*, *Lemna minor*	Immobilization; growth inhibition; altered toxicity after phototransformation.	µg–mg L^−1^ range	Acute	Germany	[49]
Cefadroxil, Cefradine	*Daphnia magna*, *Oryzias latipes*	Reduced survival; impaired reproduction and growth.	µg L^−1^–mg L^−1^	Chronic	South Korea	[50]
Ceftazidime	*Brachionus calyciflorus*	Altered feeding rate; behavioral changes during and after exposure.	µg L^−1^	Acute + post-exposure	China	[51]
Cephalexin	*Chlorella vulgaris*	Growth inhibition; reduced photosynthetic activity; partial adaptive response.	mg L^−1^	Chronic	Iran	[52]
Cefotaxime	*Danio rerio*	Increased mobility of β-lactam resistance genes in gut microbiota.	Field-realistic (µg L^−1^)	Chronic	China	[54]
Multiple cephalosporins (various dissociation forms)	*Daphnia magna*	Acute toxicity modified by phototransformation products.	µg L^−1^	Acute	China	[55]
Cephalosporin C	*Daphnia magna*	Immobilization following ionizing-radiation degradation.	mg L^−1^	Acute	China	[58]
Cefazolin (chlorination byproducts)	*Escherichia coli*	DNA damage; genotoxic effects.	mg L^−1^	Short-term	China	[56]
Ceftazidime	*Chlorella* sp.	Growth inhibition; biological stress during removal process.	mg L^−1^	Chronic	China	[53]
Cephalexin (degradation products)	Quantitative Structure–Activity Relationship (QSAR)-based models	Predicted ecotoxicity of hydroxyl-radical byproducts.	Model-based		Theoretical study	[56]

**Figure 4:**
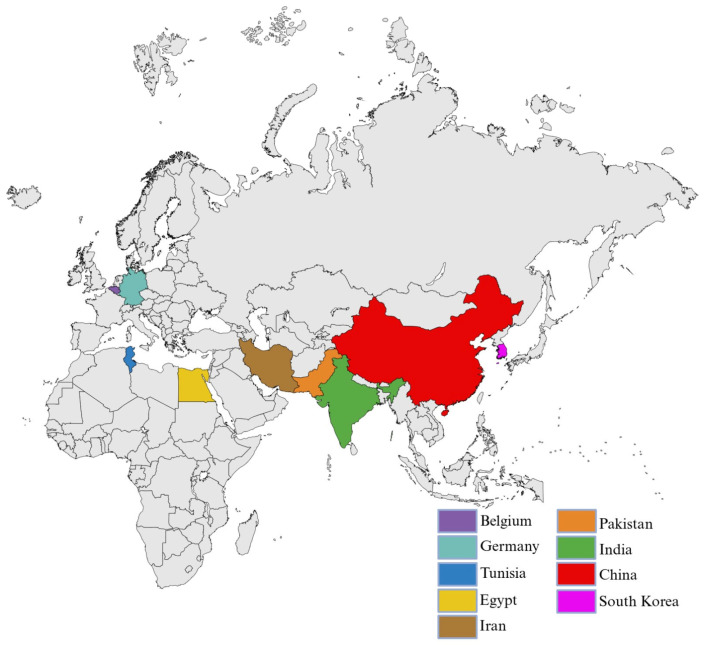
The Distribution of Cephalosporins Studies. Created in BioRender. Chelaru, I. (2026) https://BioRender.com/hn0dtts (accessed on 14 April 2026).

The studies summarized in Table 3 and Table 4 cover a wide range of experimental approaches and environmental contexts; however, their geographical distribution is not uniform. To place these findings in a broader spatial perspective, Figure 4 illustrates the geographical distribution of studies investigating the ecotoxicological effects, environmental fate, degradation, and transformation of cephalosporins in aquatic environments. This visualization helps contextualize the available evidence and highlights regional patterns and gaps in current research.

### 3.3. Fate, Transformation, and Removal Processes

The selected works cover both natural processes, such as photolysis and interactions with environmental particles, and artificial treatment options, such as photocatalysis, electrochemical degradation, advanced oxidation processes, chlorination and ionizing radiation. Table 4 presents a summary of studies on the degradation, transformation and removal of cephalosporin ABs in aquatic environments. Most experiments were performed in aqueous systems under controlled laboratory conditions, with some studies considering various water matrices to better simulate the complexity of the environment.

During the investigations, the kinetics of degradation, the transformation mechanisms and the creation of intermediate or final products were thoroughly examined. Several studies also examined the residual or modified toxicity after treatment, demonstrating that degradation does not always correspond to complete detoxification. Recent research has also found that aged microplastics can influence the transformation of cephalosporins, acting as reactive surfaces that modify photochemical processes [55,59]. In addition to modifying photochemical pathways, microplastics may influence the sorption and bioavailability of cephalosporins by acting as adsorption surfaces, potentially altering exposure dynamics and degradation rates in the surrounding water column. Sediment–water partitioning may further contribute to the persistence of cephalosporins by facilitating temporary sequestration in sediments, followed by gradual re-release into the water column; however, this process remains insufficiently characterized for most cephalosporins [15,55]. Overall, the studies summarized in Table 4 provide extensive insight into the processes governing the environmental fate of cephalosporins. Transformation products of cephalosporins are typically identified using HRMS-based approaches and proposed reaction pathways; however, their antimicrobial activity is rarely quantified, representing a major uncertainty for resistance-related risk assessment [47].

In several studies, the identification of cephalosporin transformation products revealed structural modifications primarily associated with β-lactam ring opening, side-chain cleavage, decarboxylation, and hydroxylation reactions, which may substantially alter toxicity profiles. For example, chlorination of cefazolin led to the formation of transformation products exhibiting genotoxic effects, despite substantial degradation of the parent compound, highlighting that chemical disappearance does not necessarily imply risk reduction [56]. Similarly, photochemical transformation of mixed cephalosporins resulted in photo-modified toxicity, with certain intermediates showing comparable or enhanced biological effects relative to pristine compounds [55].

Advanced oxidation and ionizing radiation treatments frequently achieved rapid parent compound degradation; however, several studies reported only partial detoxification, emphasizing the relevance of short-lived yet biologically active intermediates [52,59]. In the case of cephalosporin C, irradiation reduced acute toxicity but did not fully eliminate ecotoxicological concern, suggesting the formation of less acutely toxic but still reactive by-products [58]. Theoretical and experimental assessments of cephalexin transformation further indicated that hydroxyl radical-driven reactions can yield products with predicted residual ecotoxicity, reinforcing uncertainties in post-treatment risk assessment [57]. Recent studies indicate that aged microplastics can mediate the transformation of cephalosporins by enhancing phototransformation processes or catalyzing surface-driven degradation, leading to accelerated or structure-dependent degradation rates and potentially altered toxicity profiles, although the ecological implications of these microplastic-associated transformation pathways remain insufficiently resolved [60,61].

These findings underline the necessity of integrating transformation-product identification and toxicity assessment into environmental monitoring and treatment evaluation frameworks. From a risk-assessment perspective, reliance on parent-compound removal alone may underestimate ecological and antimicrobial-selection risks if transformation products retain toxicity or antimicrobial activity. Consequently, treatment strategies for cephalosporins should be evaluated not only based on removal efficiency but also on toxicity evolution and biological relevance of transformation products, particularly under environmentally realistic conditions.

**Table 4:** Studies on the Fate, Degradation and Transformation of Cephalosporins in Aquatic Environments.

Treatment Category	Cephalosporin(s)	Process/Technology	Aquatic Matrix	Degradation Efficiency (%)	Degradation Time	Toxicity Outcome (Parent vs. Products)	Reference
Photolysis/Natural phototransformation	Cephalosporins (multiple)	Natural photolysis	Aqueous solutions	Up to ~80–90%	Hours–days	Photo-modified toxicity observed; some products showed comparable or increased toxicity	[55]
Photocatalysis	Ceftriaxone sodium	Bi_2_WO_6_/g-C_3_N_4_ photocatalyst	Aqueous solution	>90%	Minutes–hours	Toxicity not fully eliminated; transformation products formed	[62]
Advanced oxidation processes (AOPs)	Cephalexin	Hydroxyl radical oxidation (theoretical)	Simulated aqueous system	Predicted high (>90%)	Rapid (model-based)	By-products predicted to retain residual toxicity	[57]
Advanced oxidation processes (AOPs)	β-lactam ABs (incl. cephalosporins)	UV/H_2_O_2_ or UV/persulfate	Aqueous solution	>95%	Minutes	Partial detoxification; short-lived toxic intermediates possible	[59]
Chemical oxidation	Cefazolin	Chlorination	Aqueous solution	High (>90%)	Minutes	Formation of genotoxic transformation products detected	[56]
Chemical oxidation	Cefazolin	Fe(VI)-loaded clay oxidation Metal–Organic Frameworks	Aqueous solution	Enhanced (>85–95%)	Minutes–hours	Reduced parent toxicity; by-product toxicity not fully resolved	[63]
Electrochemical treatment	Ceftazidime	(MOF)-derived CuOx-C electrode	Aqueous solution	>90%	Minutes	Effective removal; limited toxicity data on by-products	[64]
Microplastic-mediated processes	Cephalosporins (multiple)	Phototransformation enhanced by aged PS microplastics	Aqueous system with microplastics	Accelerated degradation	Depends on light exposure	Potentially altered toxicity due to modified transformation pathways	[60]
Microplastic-mediated processes	Cephalosporins (multiple)	Surface-catalyzed degradation of aged Polyvinyl chloride (PVC) microplastics	Aqueous system with microplastics	Variable	Hours–days	Structure-dependent effects; toxicity implications unresolved	[61]
Radiation-based treatment	Cephalosporin C	Ionizing radiation	Multiple water matrices	>90%	Minutes	Acute toxicity reduced; residual ecological effects possible	[58]

### 3.4. Synthesis of Review and Meta-Analyses on Cephalosporins, Antimicrobial Resistance, and One Health Implications

Other studies included in the analysis were reviews, systematic assessments and meta-analysis-based investigations to provide contextual information on the environmental relevance of cephalosporins and other β-lactam ABs. A thorough critical review synthesized the available evidence on the occurrence, fate, ecotoxicity and removal technologies of cephalosporins in aquatic environments, highlighting their widespread detection in surface waters and wastewaters and identifying major knowledge gaps related to transformation products and long-term ecological risks [15].

Other systematic reviews have expanded the scope to include antibiotic residues from livestock, wastewater, and soils on a global scale, highlighting environmental dissemination pathways and the role of agricultural practices [65]. Studies of β-lactamase-resistant bacteria, such as carbapenemases and extended-spectrum β-lactamase (ESBL)-producing Enterobacteriales, have found resistant strains in food-producing animals, wildlife, aquatic systems, and consumer food products, highlighting the interconnected nature of environmental and human health risks [66,67].

Importantly, these investigations have consistently identified ESBL genes strongly associated with resistance to third-generation cephalosporins, particularly *blaCTX-M*, *blaSHV*, and *blaTEM*, as the most frequently detected resistance determinants in wastewater-impacted aquatic environments [68,69]. These genes confer reduced susceptibility to cephalosporins such as cefotaxime, ceftazidime, and ceftriaxone and are widely considered molecular indicators of cephalosporin-driven selective pressure in surface waters, sediments, and effluents [70,71].

Several studies have adopted a “One Health” approach, integrating environmental monitoring with genomic and microbiological analysis. These studies have identified Escherichia coli, a World Health Organization (WHO) critical priority bacterium, and other resistant bacteria in water systems, aquaculture products, and wastewater-impacted environments, frequently linking environmental exposure to public health concerns [39,72]. Genomic surveillance has further revealed that cephalosporin-associated resistance genes are often located on mobile genetic elements, facilitating horizontal transfer between environmental and clinically relevant bacteria [73,74].

Recent research has examined the influence of treated wastewater on aquatic biofilms, revealing that both abiotic and biotic variables contribute to the formation of environmental resistomes and help identify novel resistance mechanisms [75]. Such biofilm-associated resistomes are frequently enriched in ESBL genes linked to cephalosporin exposure, emphasizing the role of treated effluents as reservoirs and mixing points for resistance evolution [34,76]. Together, these results highlight the necessity to combine compound-specific chemical monitoring of cephalosporins with microbiological and genetic techniques targeting cephalosporin-associated resistance genes, in order to better understand the development, persistence, and dissemination of resistance in aquatic environments.

### 3.5. Limitations and Future Perspectives

Despite the growing body of literature on cephalosporins in aquatic environments, several critical knowledge gaps persist, which constrain comprehensive environmental risk assessments and the development of effective management strategies. Environmental parameters such as pH, salinity, and natural organic matter are expected to strongly influence cephalosporin stability and degradation kinetics; however, comparative data between aquaculture ponds and freshwater systems remain limited.

One of the most significant gaps concerns the limited availability of long-term, field-based monitoring studies. Although many studies have documented the presence of cephalosporins in surface waters, effluents, and sediments, most of these investigations are based on short-term sampling efforts, often limited to regions or seasons. This constrains the comprehension of temporal variability, long-term exposure scenarios, and the impact of climatic variables on the distribution and persistence of cephalosporins [45,77].

Another notable gap concerns the environmental relevance of ecotoxicological assessments. Most toxicity studies have been conducted under controlled laboratory conditions, using concentrations that may exceed those typically observed in natural waters. Although these studies provide valuable mechanistic insights, they may not adequately reflect the chronic low-dose exposure conditions encountered by aquatic organisms in their natural environment. Furthermore, ecotoxicity tests have focused on a small number of model organisms, such as Daphnia magna and microalgae, with little research examining higher trophic levels, community-level effects, or multigenerational consequences [49,50].

Transformation products represent another unexplored aspect of cephalosporin contamination. Emerging research suggests that photolysis, chlorination, and advanced oxidation processes can produce byproducts with different or even higher toxicity than the parent molecules [55,58]. However, most environmental monitoring programs exclude transformation products due to analytical issues and lack of reference standards. Combining non-targeted screening methods with effect-directed analysis could significantly improve the detection and prioritization of hazardous transformation products. Compared to other β-lactam antibiotics, specific knowledge gaps for cephalosporins persist, particularly regarding their environmental stability, transformation-product toxicity, and resistance-selection dynamics under environmentally realistic exposure conditions. While cephalosporins are frequently detected at ng–µg L^−1^ levels, direct links between environmental concentrations and clinical resistance development remain difficult to establish due to complex exposure pathways and co-selection processes [15]. Future research should prioritize environmentally relevant cephalosporins such as cefotaxime, ceftazidime, and ceftriaxone, which are frequently detected in wastewater-impacted systems and closely associated with extended-spectrum β-lactamase resistance, with particular emphasis on aquaculture-adjacent environments, sediment interactions, and microbiome-level responses.

The interaction of cephalosporins with concomitant environmental stressors requires further investigation. Recent studies have shown that microplastics can affect the degradation and toxicity of cephalosporins by providing reactive surfaces and altering photochemical pathways [55]. However, research on the combined impact of ABs, microplastics, metals, and natural organic matter is scarce. Such interactions are anticipated to influence the fate and ecological impact of cephalosporins in aquatic environments.

Technologically, improved treatment procedures hold promise for the removal of cephalosporins, but their broader environmental impacts have not yet been fully assessed. Many studies focus on high removal efficiencies without considering the development of hazardous intermediates or the potential promotion of antibiotic resistance genes during treatment. In addition, the scalability and applicability of these advanced treatment technologies to aquaculture settings remain insufficiently evaluated, particularly under field-realistic conditions. Future research should utilize integrated assessment frameworks that include chemical analysis, ecotoxicological testing, and resistance-related endpoints to ensure that treatment options reduce risk rather than convert pollutants [54,61].

In addition to advanced physical and chemical treatment technologies, future studies should increasingly explore the potential of natural sorbents derived from plant and animal waste materials as sustainable remediation options for cephalosporins in aquatic environments. Biomass-based substrates such as parsley residues, wood chips, and other agricultural or food-processing by-products have attracted attention due to their availability, low cost, renewability, and favorable surface chemistry for antibiotic adsorption. Such materials may offer an eco-friendly alternative for the removal of cephalosporins, their metabolites, and transformation products, particularly in decentralized or low-resource treatment settings. Moreover, the integration of natural sorbents into existing treatment systems could contribute to circular-economy strategies by valorizing organic waste streams while reducing pharmaceutical pollution. However, further research is required to evaluate their removal efficiency under environmentally realistic conditions, regeneration potential, and long-term performance. In addition to advanced treatment technologies, future studies should increasingly explore the potential of natural sorbents derived from plant and animal waste materials, such as parsley residues and wood chips, as sustainable remediation options for cephalosporins in aquatic environments [78,79,80,81].

Finally, addressing cephalosporin pollution in aquatic habitats requires a more comprehensive, “One Health” approach that recognizes the interdependence of environmental, animal, and human health. The presence of cephalosporin and β-lactam resistance drivers in water bodies, aquatic beings, and food chains indicates that environmental reservoirs may contribute to the global epidemic of antimicrobial resistance [54,82]. Coordinated surveillance efforts, coordinated analytical techniques, and cross-sectoral policy frameworks will be essential to minimize long-term hazards and protect both the environment and public health. By focusing specifically on cephalosporins, this review highlights challenges and research priorities that are often overlooked in broader antibiotic assessments.

## 4. Conclusions

This review provides a thorough examination of the occurrence, environmental fate, ecotoxicological consequences and removal of cephalosporin ABs in aquatic environments. Cephalosporins have been found in a wide range from low ng L^−1^ levels in surface waters to several µg L^−1^ in hospital and municipal wastewater effluents, with detection frequencies often exceeding 50–80% in wastewater-impacted systems, indicating continuous environmental inputs. Their frequent detection highlights the limitations of traditional wastewater treatment techniques, as well as the ongoing presence of these chemicals in aquatic environments.

In aquatic systems, cephalosporins undergo a variety of transformation pathways, which are influenced by both natural processes, such as photolysis, and developed treatment technologies, such as enhanced oxidation, photocatalysis, and electrochemical degradation. Although many of these processes achieve high parent-compound removal efficiencies, degradation frequently results in the formation of transformation products with altered physicochemical properties and, in certain cases, comparable or increased toxicity. Consequently, removal of the parent compound does not necessarily imply complete detoxification or ecological risk reduction.

Ecotoxicological studies show that cephalosporins and their transformation products can induce adverse effects at different trophic levels, affecting aquatic invertebrates, algae, macrophytes, fish and microbial communities. Sublethal effects—such as growth inhibition, behavioral disturbances, and shifts in microbial composition—have been reported at environmentally relevant concentrations, raising concerns regarding chronic exposure. In addition, increasing evidence links cephalosporin contamination to the dissemination of β-lactam resistance genes, reinforcing the role of aquatic environments as both reservoirs and transmission pathways for antimicrobial resistance.

Beyond these scientific findings, the results have important regulatory and management implications. Currently, specific discharge limits for cephalosporins and their transformation products are largely absent from wastewater regulations, despite their frequent detection and potential to promote antimicrobial resistance. Future regulatory frameworks should therefore consider updated effluent standards that explicitly account for both parent compounds and transformation products, integrate antimicrobial resistance surveillance into routine water-quality monitoring programs, and prioritize the adoption of advanced treatment technologies at high-risk discharge points, particularly hospitals and pharmaceutical manufacturing facilities. Such measures are essential to translate emerging scientific evidence into effective environmental protection and public-health strategies.

## Figures and Tables

**Table 1:** Classification of Cephalosporins [22].

Cephalosporin(s)	Examples	Activity	Half-Life (Human Plasma)	Environmental Half-Life/Stability
First-generation	Cefalotin, cefaloridin, cephalexin, cephapirin, cefazolin, cefadroxil, cephradine and others.	Staphylococci, streptococci, pneumococci and many types of enterobacteria.	~0.5–2 h [23]	Low–moderate stability; rapid degradation in surface waters (hours–days), sensitive to hydrolysis and photolysis [24]
Second-generation	Cefuroxime, cefamandole, cefoxitin, cefotetan, cefaclor, and others.	Gram-positive microorganisms resistant to the action of beta-lactamases.	~1–2 h [23]	Moderate stability; degradation typically within days under light and oxidative conditions [24,25]
Third generation	Cefotaxime, ceftizoxime, ceftriaxone, ceftazidime, cefoperazone, and many others.	Enterobacteria, including those resistant to ABs. Moderately active staphylococci.	~1–8 h (ceftriaxone up to ~6–8 h), [23,26]	Moderate–high persistence; frequently detected in wastewater effluents and receiving waters (days–weeks), incomplete removal in WWTPs [15,24]
Fourth generation	Cefepime and cefpirome.	Broad spectrum of Gram-positive and Gram-negative aerobes.	~2–3 h [23]	Moderate stability; susceptible to photochemical and oxidative degradation, limited field data available [24]

**Table 2:** Analytical Methods and Occurrence of Cephalosporins in Aquatic Environments.

Study Focus	Type of Antibiotic	Matrix	Key Findings	Country	References
Quantification of cephalosporins by LC–MS/MS	16 cephalosporins	Surface water, wastewater	Sensitive and selective LC-MS/MS method that allows simultaneous quantification of multiple cephalosporins at trace levels.	Egypt	[42]
Determination of multi-class ABs by Solid Phase Extraction-Liquid Chromatography-Electrospray Ionization-Mass Spectrometry (SPE–LC–ESI–MS)	Broad-spectrum ABs (including cephalosporins)	Surface water	Validated Solid Phase Extraction–Liquid Chromatography–Mass Spectrometry (SPE–LC–MS) method suitable for environmental monitoring of ABs.	India	[43]
Occurrence and distribution of ABs and Antibiotic Resistance Gene (ARGs)	Multiple ABs	Groundwater, surface water, sediment	Widespread detection of ABs with significant correlations to antibiotic resistance genes.	China	[17]
Occurrence and fate in wastewater treatment systems	Human ABs	Influent, effluent, receiving river	Partial removal during treatment; persistence in effluent-receiving river.	India	[44]
Riverine occurrence and ecological risk	Multiple ABs	River water	High detection frequency: ecological risk identified for selected compounds.	China	[45]
Occurrence and risk in urban rivers	Selected ABs	Urban river water	ABs detected at µg L^−1^ levels; potential ecotoxicological and AMR risks.	Pakistan	[46]
High-resolution multi-class screening	46 antimicrobial residues	Pond water	Ultra-High-Performance Liquid Chromatography (UHPLC)–Orbitrap–High-Resolution Mass Spectrometer (HRMS) enabled comprehensive screening of antimicrobial residues.	Belgium	[47]
Optimized Solid Phase Extraction-Liquid Chromatography-Tandem Mass Spectrometry (SPE–LC–MS/MS method)	ABs (multi-class)	Groundwater, surface water, treated water	Improved recoveries and low detection limits within water matrices.	Iran	[38]
Occurrence and removal in hospital wastewater	ABs and ARGs	Hospital wastewater	Incomplete removal of ABs and resistance genes during treatment.	China	[31]
Persistence of multidrug-resistant bacteria	Antibiotic-resistant Enterobacterales	Urban, industrial, surface water	High persistence of resistant bacteria linked to contaminated water bodies.	Tunisia	[48]

## Data Availability

No new data were created or analyzed in this study. Data sharing is not applicable to this article.

## Abbreviations

The following abbreviations are used in this manuscript:

ABs	Antibiotics
AMR	Antimicrobial resistance
AOPs	Advanced oxidation processes
ARGs	Antibiotic Resistance Gene
ESBL	Extended-spectrum β-lactamase
HPLC	High-performance liquid chromatography
HRMS	High-Resolution Mass Spectrometer
LC-MS/MS	Liquid chromatography-mass spectrometry
MOF	Metal–Organic Frameworks
PVC	Polyvinyl chloride
QSAR	Quantitative Structure–Activity Relationship
SPE	Solid phase extraction
SPE–LC–ESI–MS	Solid Phase Extraction–Liquid Chromatography–Mass Spectrometry
SPE–LC–MS/MS	Solid Phase Extraction–Liquid Chromatography–Tandem Mass Spectrometry
UHPLC	Ultra-High-Performance Liquid Chromatography
WHO	World Health Organization
WWTPs	Wastewater treatment plants
